# Case Report: Malignant colonic PEComa presenting as fever of unknown origin with spontaneous perforation

**DOI:** 10.3389/fonc.2026.1852776

**Published:** 2026-05-20

**Authors:** Gizem Gunes, Adem Ozcan

**Affiliations:** Department of Surgical Oncology, Ankara Bilkent City Hospital, Ankara, Türkiye

**Keywords:** colon, fever of unknown origin, mesenchymal tumor, PEComa, perforation

## Abstract

**Background:**

Perivascular epithelioid cell tumors (PEComas) are rare mesenchymal neoplasms with variable malignant potential, and gastrointestinal involvement is particularly uncommon. Colonic PEComas are exceedingly rare and may present with nonspecific symptoms, complicating early diagnosis.

**Case presentation:**

A 58-year-old male presented with prolonged fever, fatigue, and weight loss. Laboratory findings revealed leukocytosis and anemia, while infectious and rheumatologic evaluations were unremarkable. Following rectal bleeding, colonoscopy identified a perforated lesion in the sigmoid colon. Imaging demonstrated extensive tumoral wall thickening, and the patient underwent emergency surgery due to acute abdomen. Histopathological and immunohistochemical analysis confirmed malignant PEComa. The patient received adjuvant chemotherapy and radiotherapy and remained disease-free at 5-year follow-up.

**Conclusion:**

This case highlights an unusual presentation of colonic PEComa with fever of unknown origin and spontaneous perforation. Clinicians should consider rare malignancies in patients with unexplained inflammatory syndromes, particularly when accompanied by gastrointestinal findings.

## Introduction

Perivascular epithelioid cell tumors (PEComas) represent a rare group of mesenchymal neoplasms characterized by distinctive perivascular epithelioid cells exhibiting dual melanocytic and smooth muscle differentiation. Owing to their rarity and histopathological overlap with other mesenchymal and melanocytic tumors, PEComas pose significant diagnostic challenges, often requiring comprehensive immunohistochemical analysis for definitive identification (1, 2).

The biological behavior of PEComas is highly heterogeneous, ranging from benign lesions to aggressive malignancies with metastatic potential. Although several histopathological features—such as tumor size, mitotic activity, necrosis, and vascular invasion—have been proposed as indicators of malignancy, universally accepted criteria for risk stratification and management remain lacking (2, 3). Consequently, treatment strategies are largely guided by limited case series and expert opinion.

Gastrointestinal involvement of PEComas is uncommon; however, it represents a recognized site of disease occurrence. Within the gastrointestinal tract, the colorectum—particularly the colon—has been reported among the relatively more frequently affected anatomical locations. Reported cases typically present with nonspecific gastrointestinal symptoms, including abdominal pain or rectal bleeding, which may further complicate early diagnosis (1, 4). In the largest clinicopathological series to date, Doyle et al. highlighted the marked heterogeneity in presentation, morphology, and clinical behavior of gastrointestinal PEComas, underscoring the challenges in diagnosis and risk stratification (1).

Importantly, atypical clinical manifestations such as fever of unknown origin (FUO) or tumor perforation have been rarely described in association with colonic PEComas, posing additional diagnostic and therapeutic challenges.

In this report, we present a rare case of malignant PEComa of the sigmoid colon presenting with prolonged fever of unknown origin and spontaneous tumor perforation, highlighting the diagnostic complexity and the need for heightened clinical awareness in patients with unexplained systemic inflammatory symptoms.

## Case presentation

A 58-year-old male patient was admitted to the infectious diseases department with a one-month history of persistent high fever, fatigue, and unintentional weight loss. The patient had no known chronic medical conditions, no prior abdominal surgery, and was not taking any regular medications.

There was no known family history of malignancy or hereditary cancer syndromes. In particular, no clinical features suggestive of tuberous sclerosis complex (TSC) were identified. The patient had no history of smoking or alcohol abuse, and no significant occupational or environmental exposures were reported. Genetic testing for TSC1/TSC2 mutations was not performed.

Physical examination at admission revealed a body temperature of 38.5 °C, heart rate of 105 beats per minute, and blood pressure of 110/70 mmHg. The patient appeared fatigued but was hemodynamically stable. Abdominal examination demonstrated localized tenderness in the lower abdomen, particularly in the left lower quadrant, with mild guarding. There were no signs of generalized peritonitis at initial presentation. No significant abdominal distension was observed. Other systemic examination findings were unremarkable.

Initial laboratory evaluation revealed leukocytosis (white blood cell count: 16,000/mm³ with 80% neutrophils), anemia (hemoglobin: 10.4 g/dL), and elevated inflammatory markers (C-reactive protein: 20 mg/L), while procalcitonin was 0.4 ng/mL. Extensive infectious and rheumatologic investigations, including blood cultures and autoimmune panels, were unremarkable.

Given the combination of persistent fever and elevated inflammatory markers, an infectious etiology was initially suspected; however, extensive microbiological investigations, including blood cultures, remained negative. Rheumatologic and autoimmune causes were also considered but were not supported by laboratory findings. The subsequent development of anemia and rectal bleeding raised concern for an underlying gastrointestinal pathology, prompting endoscopic evaluation.

**Figure 1:**
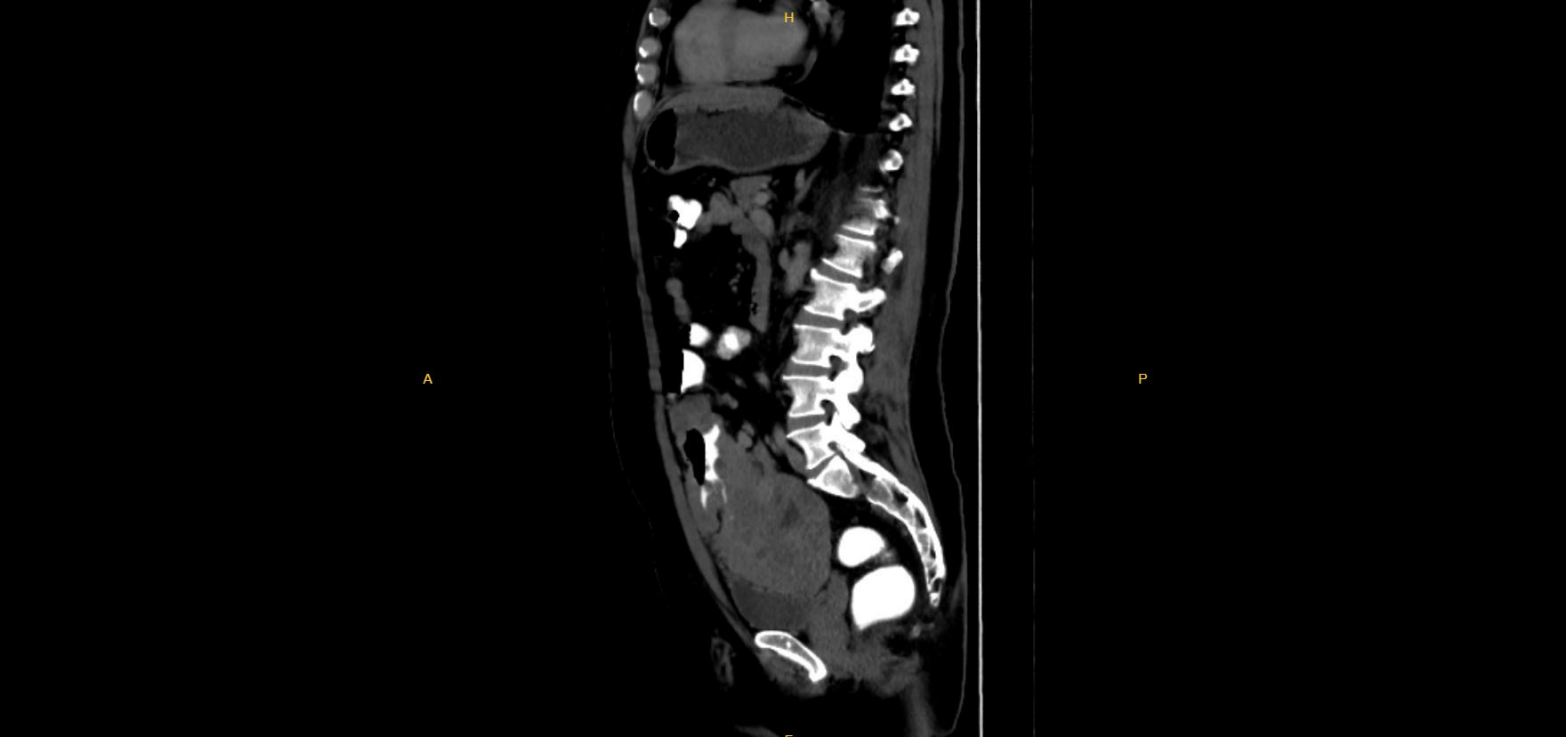
Contrast-enhanced CT showing sigmoid colon wall thickening.

Due to the presence of anemia and newly developed rectal bleeding, gastrointestinal evaluation was undertaken. Colonoscopy revealed a perforated lesion approximately 2 cm in diameter located in the sigmoid colon, 55 cm from the anal verge. Following this finding, contrast-enhanced abdominal computed tomography (CT) was performed, demonstrating an approximately 18 cm segment of irregular, nodular wall thickening in the sigmoid colon, with a maximum thickness of 47 mm, highly suspicious for an underlying malignant neoplasm (**Figure 1**).

**Figure 2:**
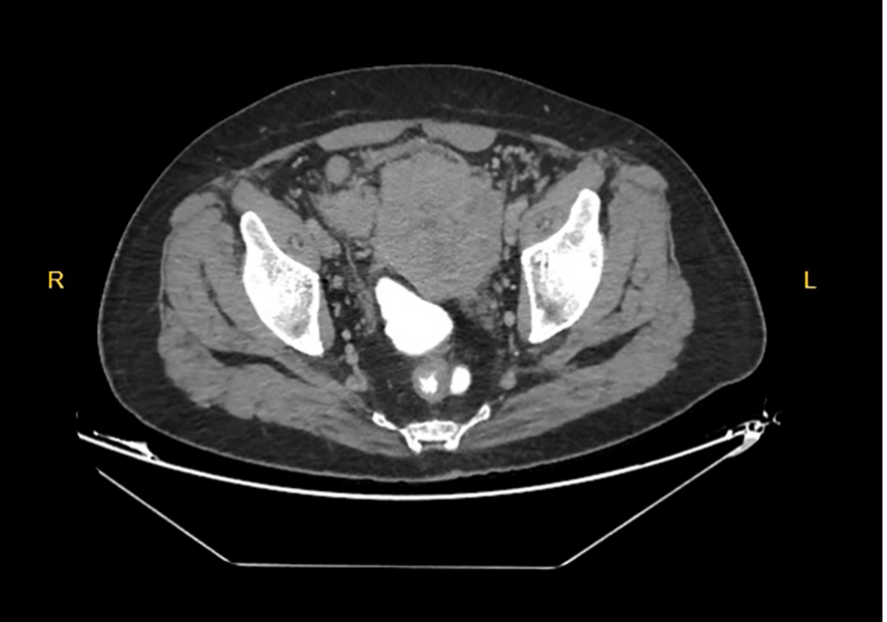
Contrast-enhanced CT image showing nodular thickening and suspected focal perforation of the sigmoid colon.

Given the clinical presentation of acute abdomen, the patient underwent emergency surgery. Intraoperatively, a large mass originating from the sigmoid colon was identified, with multiple contained perforations extending toward the anterior and lateral abdominal walls (**Figure 2**). A sigmoid colon resection with Hartmann’s procedure was performed. Gross examination revealed a 16 × 12 cm tumor with extension to the mesenteric margin. Resection margins were negative except for involvement at the mesenteric surgical margin. A total of 15 lymph nodes were examined, of which 1 showed metastatic involvement. Lymphovascular invasion was present.

Histopathological examination revealed a malignant mesenchymal tumor composed of epithelioid cells with marked atypia. According to the World Health Organization classification and Folpe criteria, the tumor demonstrated several high-risk features, including large tumor size (>5 cm), infiltrative growth pattern, lymphovascular invasion, and increased mitotic activity, supporting a diagnosis of malignant PEComa. Mitotic activity was quantified as 7 mitoses per 50 high-power fields, including atypical forms, and tumor necrosis was present. The tumor demonstrated multiple high-risk features according to the Folpe criteria, as detailed in **Table 1**.

**Table 1:** Folpe risk stratification criteria and corresponding findings in the present case.

Criterion	Presence	Evidence in the present case
Tumor size >5 cm	Present	Tumor measured 16 × 12 cm
Infiltrative growth pattern	Present	Tumor infiltrating surrounding tissues and extending to mesenteric margin
Mitotic activity	Present	7 mitoses per 50 high-power fields with atypical mitotic figures
Tumor necrosis	Present	Areas of tumor necrosis identified histologically
Lymphovascular invasion	Present	Tumor cells identified within vascular channels

Based on these findings, the tumor fulfills multiple high-risk features according to the Folpe criteria, strongly supporting its classification as malignant PEComa.

Immunohistochemical analysis showed focal positivity for HMB-45 and Melan-A, while S100 and SOX10 were negative, excluding melanoma and related entities. Additional markers including epithelial, myogenic, and hematological lineage markers were negative, ruling out differential diagnoses such as carcinoma, rhabdomyosarcoma, and hematologic malignancies. The tumor also demonstrated diffuse vimentin positivity and focal expression of CD99, desmin, and CD56. The Ki-67 proliferation index was approximately 10–15%. The immunohistochemical findings are summarized in **Table 2**.

**Table 2:** Immunohistochemical profile of the tumor.

Marker	Result	Interpretation
HMB-45	Focal positive	Supports PEComa (melanocytic differentiation)
Melan-A	Focal positive	Consistent with PEComa
S100	Negative	Argues against melanoma
SOX10	Negative	Excludes melanocytic neoplasms
Vimentin	Positive	Mesenchymal origin
Desmin	Focal positive	Supports myogenic differentiation
CD99	Focal positive	Nonspecific finding
CD56	Focal positive	Nonspecific finding
Ki-67	10–15%	Moderate proliferative activity

The patient underwent sigmoid colon resection with Hartmann’s procedure for a sigmoid colon–localized PEComa with positive surgical margins (CS+). Given the presence of high-risk pathological features, adjuvant treatment was initiated. The patient received six cycles of ifosfamide, mesna, and doxorubicin (IMA) chemotherapy. This was followed by adjuvant radiotherapy to the pelvic tumor bed, delivered at a total dose of approximately 50–54 Gy in conventional fractions.

Given the absence of molecular profiling for TSC1/TSC2 alterations and the presence of multiple high-risk pathological features, a conventional soft tissue sarcoma–based chemotherapy regimen was selected. Targeted therapies, including mTOR inhibitors, were considered potential options in the setting of disease progression or recurrence.

Post-treatment follow-up included periodic contrast-enhanced imaging and colonoscopic evaluation. Imaging was performed every 3–6 months during the first year and annually thereafter. Colonoscopic surveillance was conducted during follow-up, with no evidence of local recurrence. Clinician-assessed outcomes demonstrated no radiological or endoscopic evidence of recurrence or metastasis throughout the follow-up period. The patient remained asymptomatic, with no recurrence of fever, gastrointestinal bleeding, or weight loss. Treatment was generally well tolerated, with no significant chemotherapy- or radiotherapy-related adverse events reported. At 5-year follow-up, the patient remained disease-free.

The coexistence of prolonged fever of unknown origin and spontaneous tumor perforation significantly complicated the diagnostic process and contributed to delayed recognition of the underlying malignancy.

The clinical course of the patient is summarized in the timeline **Table 3**.

**Table 3:** Clinical timeline of the patient from symptom onset to 5-year follow-up.

Time point	Clinical event
1 month before admission	Onset of persistent fever, fatigue, and weight loss
Day 0 (Admission)	Hospital admission; initial clinical and laboratory evaluation
Days 1–7	Extensive infectious and rheumatologic workup (negative)
Day 7	Development of rectal bleeding
Day 8	Colonoscopy: perforated sigmoid lesion detected
Day 9	Contrast-enhanced CT: long segment tumoral wall thickening
Day 10	Emergency surgery (Hartmann’s procedure)
Postoperative period	Histopathological diagnosis of malignant PEComa
4 weeks postoperatively	Initiation of adjuvant chemotherapy
Months 1–4	Completion of 6 cycles of chemotherapy
Months 5–6	Adjuvant radiotherapy
6-month follow-up	No evidence of recurrence or metastasis
12-month follow-up	Colostomy closure
5-year follow-up	Disease-free survival confirmed

## Discussion

Perivascular epithelioid cell tumors (PEComas) represent a rare and heterogeneous group of mesenchymal neoplasms characterized by distinctive morphologic and immunophenotypic features, yet their clinical course remains highly unpredictable. Gastrointestinal involvement is uncommon, and colonic localization constitutes a rare entity within this already limited spectrum, with only a small number of cases reported to date (1). This rarity significantly limits the development of standardized diagnostic and therapeutic algorithms.

The diagnosis of PEComa remains particularly challenging due to its morphological overlap with other mesenchymal and melanocytic neoplasms, including gastrointestinal stromal tumors (GIST) and malignant melanoma. Therefore, immunohistochemical evaluation plays a pivotal role in establishing the diagnosis. The co-expression of melanocytic markers such as HMB-45 and Melan-A, together with variable expression of smooth muscle markers, is considered a defining feature of PEComas (1, 2). In the present case, the absence of S100 and SOX10 expression was crucial in excluding melanoma and related entities, reinforcing the importance of a comprehensive immunophenotypic panel in the differential diagnosis. In addition, the differential diagnosis included gastrointestinal stromal tumors and other mesenchymal malignancies; however, the immunohistochemical profile and morphological features were not compatible with these entities.

The biological behavior of PEComas is highly variable, ranging from benign lesions to aggressive malignancies with metastatic potential. Folpe et al. proposed a set of histopathological criteria for risk stratification, including tumor size greater than 5 cm, infiltrative growth pattern, high mitotic activity, necrosis, and vascular invasion (2). Our case fulfilled several of these high-risk features, including large tumor size and lymphovascular invasion, supporting its classification as malignant PEComa. Subsequent analyzes have validated the prognostic relevance of these criteria, although their universal applicability remains limited due to the rarity of the disease and the lack of large-scale studies (3, 5).

Clinically, gastrointestinal PEComas typically present with nonspecific symptoms such as abdominal pain, gastrointestinal bleeding, or are incidentally detected during imaging or endoscopic evaluation (1, 4). In contrast, our patient presented with prolonged fever of unknown origin (FUO), which is an exceedingly rare manifestation. Systemic inflammatory presentations in PEComas have only been sporadically described in the literature, and their pathophysiological basis remains unclear, with proposed mechanisms including tumor necrosis and cytokine-mediated inflammatory responses (6). This atypical clinical course may delay diagnosis and complicate management, highlighting the need for heightened clinical suspicion. In addition, rare case reports of colonic PEComa, including that by Slice et al., have described unusual clinical presentations and aggressive local behavior, further emphasizing the heterogeneous nature of this disease (7).

Another striking feature of our case is tumor perforation, which is rarely reported in colonic PEComas. While perforation is more commonly associated with advanced colorectal adenocarcinoma or aggressive sarcomas, its occurrence in PEComa underscores the potential for aggressive biological behavior in selected cases. The coexistence of FUO and spontaneous perforation makes this presentation particularly unusual and clinically relevant. To our knowledge, reports describing this combination are extremely limited, further emphasizing the uniqueness of our case and its contribution to the current literature (6–8).

Surgical resection remains the cornerstone of treatment for localized PEComas and is currently considered the only curative option (3). However, due to the absence of standardized treatment guidelines, the role of adjuvant therapy remains controversial. Recent advances in molecular characterization have identified alterations in the TSC1/TSC2 genes, leading to activation of the mTOR signaling pathway in a subset of PEComas (3, 9). This has provided a rationale for the use of mTOR inhibitors such as sirolimus and everolimus, particularly in advanced or metastatic disease. Emerging evidence from recent studies published after 2020 suggests that targeted therapies may offer promising outcomes in selected patients, although prospective data remain limited (9, 10). In addition, recent clinicopathological evidence focusing specifically on colorectal PEComas has further emphasized the heterogeneity of treatment outcomes; in a series of seven cases, Ma et al. reported that prognosis is closely associated with tumor size, mitotic activity, and other high-risk features, underscoring the importance of individualized management and careful long-term follow-up (11).

In the present case, given the presence of multiple high-risk pathological features and the lack of molecular profiling, a conventional soft tissue sarcoma–based chemotherapy regimen was administered, followed by radiotherapy. Notably, the patient achieved long-term disease-free survival at 5 years of follow-up. This favorable outcome, despite high-risk features, highlights the potential benefit of aggressive multimodal treatment in carefully selected patients and underscores the importance of individualized management strategies in the absence of consensus guidelines.

Given the rarity of PEComas and the lack of robust prospective data, current knowledge is largely derived from case reports and small case series. Therefore, each additional well-documented case plays a critical role in expanding the understanding of disease behavior, prognostic factors, and optimal treatment approaches. Taken together, the morphological features in combination with the immunohistochemical profile supported the diagnosis of malignant PEComa.

This case has several strengths. It describes a rare clinical presentation combining fever of unknown origin, colonic malignant PEComa, and spontaneous tumor perforation. The diagnosis was supported by consistent morphological and immunohistochemical findings, including focal positivity for HMB-45 and Melan-A and negativity for S100 and SOX10. In addition, comprehensive infectious and rheumatologic evaluations were performed and were unremarkable, strengthening the diagnostic process. The long-term disease-free follow-up further supports the clinical relevance of this case.

However, several limitations should be acknowledged. As a single-case report, the findings are inherently limited in generalizability. Molecular analysis for TSC1/TSC2 alterations was not performed, which may have provided additional insight into targeted therapeutic options. Although extensive investigations were conducted, fever of unknown origin may have multifactorial causes, and a direct causal relationship between systemic inflammatory findings and tumor biology cannot be definitively established. Furthermore, although imaging and surgical findings were consistent with advanced disease, detailed staging data were limited.

Our case broadens the clinical spectrum of colonic PEComas by demonstrating an unusual presentation with FUO and tumor perforation, emphasizing that rare intra-abdominal malignancies should be considered in the differential diagnosis of unexplained systemic inflammatory syndromes, particularly when accompanied by gastrointestinal findings.

## Conclusion

Colonic PEComas remain diagnostically and therapeutically challenging due to their rarity and unpredictable clinical course. This case demonstrates that atypical presentations, such as fever of unknown origin and spontaneous perforation, may obscure an underlying aggressive malignancy and delay diagnosis.

Our findings emphasize that PEComa should be considered in the differential diagnosis of unexplained inflammatory syndromes, particularly when accompanied by gastrointestinal findings. Given the absence of standardized treatment strategies, accumulation of well-documented cases is essential to improve risk stratification and guide future therapeutic approaches.

## Data Availability

The original contributions presented in the study are included in the article/supplementary material. Further inquiries can be directed to the corresponding author.
